# Multimodal imaging of hair follicle bulge-derived stem cells in a mouse model of traumatic brain injury

**DOI:** 10.1007/s00441-020-03173-1

**Published:** 2020-02-08

**Authors:** Timo Schomann, Juvita D. Iljas, Ivo Que, Yuedan Li, Ernst Suidgeest, Luis J. Cruz, Johan H.M. Frijns, Alan Chan, Clemens M.W.G. Löwik, Margriet A. Huisman, Laura Mezzanotte

**Affiliations:** 1 Department of Otorhinolaryngology and Head & Neck Surgery, Leiden University Medical Center, Leiden, the Netherlands; 2 Percuros B.V, Leiden, the Netherlands; 3 Max Planck Institute for Metabolism Research, Cologne, Germany; 4 Translational Nanobiomaterials and Imaging, Department of Radiology, Leiden University Medical Center, Leiden, the Netherlands; 5 Department of Radiology, Leiden University Medical Center, Leiden, the Netherlands; 6 Leiden Institute for Brain and Cognition, Leiden University, Leiden, the Netherlands; 7 Optical Molecular Imaging, Department of Radiology and Nuclear Medicine, Erasmus Medical Center, Rotterdam, the Netherlands; 8 Department of Molecular Genetics, Erasmus Medical Center, Rotterdam, the Netherlands; 9 Hair Science Institute, Maastricht, the Netherlands

**Keywords:** Hair follicle bulge-derived stem cells, Bioluminescence imaging, Magnetic resonance imaging, Brain injury, Stem cell treatment, Tracking

## Abstract

Traumatic brain injury (TBI) is a devastating event for which current therapies are limited. Stem cell transplantation may lead to recovery of function via different mechanisms, such as cell replacement through differentiation, stimulation of angiogenesis and support to the microenvironment. Adult hair follicle bulge-derived stem cells (HFBSCs) possess neuronal differentiation capacity, are easy to harvest and are relatively immune-privileged, which makes them potential candidates for autologous stem cell-based therapy. In this study, we apply in vivo multimodal, optical and magnetic resonance imaging techniques to investigate the behavior of mouse HFBSCs in a mouse model of TBI. HFBSCs expressed Luc2 and copGFP and were examined for their differentiation capacity in vitro. Subsequently, transduced HFBSCs, preloaded with ferumoxytol, were transplanted next to the TBI lesion (cortical region) in nude mice, 2 days after injury. Brains were fixed for immunohistochemistry 58 days after transplantation. Luc2- and copGFP-expressing, ferumoxytol-loaded HFBSCs showed adequate neuronal differentiation potential in vitro*.* Bioluminescence of the lesioned brain revealed survival of HFBSCs and magnetic resonance imaging identified their localization in the area of transplantation. Immunohistochemistry showed that transplanted cells stained for nestin and neurofilament protein (NF-Pan). Cells also expressed laminin and fibronectin but extracellular matrix masses were not detected. After 58 days, ferumoxytol could be detected in HFBSCs in brain tissue sections. These results show that HFBSCs are able to survive after brain transplantation and suggest that cells may undergo differentiation towards a neuronal cell lineage, which supports their potential use for cell-based therapy for TBI.

**Electronic supplementary material:**

The online version of this article (10.1007/s00441-020-03173-1) contains supplementary material, which is available to authorized users.

## Introduction

In recent years, stem cell therapy has attracted huge interest as a new therapeutic method for the treatment of brain injury. Many studies using animal models and even human clinical trials have demonstrated the potential of stem cell transplantation for the treatment of neurological disorders (Hasan et al. 2017; Lemmens and Steinberg 2013). The goal of stem cell therapy is the formation of new tissue to replace damaged tissue by utilizing the regenerative capacity of stem cells (Kiasatdolatabadi et al. 2017).

Application of autologous stem cells, such as bone marrow-derived mesenchymal stromal cells (BM-MSCs) and human umbilical cord blood cells, could induce neuro-restorative effects in the brain after injury (Bang et al. 2016; Caplan 2017). In general, these effects are mainly attributed to paracrine mechanisms such as the stimulatory effect of stem cells on endogenous cells to release growth and trophic factors. Mesenchymal stromal cells have the ability to migrate (Ngen et al. 2015), differentiate in neural precursor cells in vitro (Alexanian et al. 2008) and contribute to neuronal repair due to their immunomodulatory properties and other mechanisms (Li and Chopp 2009; Munoz et al. 2005; Zanier et al. 2014; Zhao et al. 2016). The advantage of BM-MSCs is that they can be harvested from the patient allowing autologous stem cell therapy. Furthermore, the latter allows the conduction of clinical trials using BM-MSCs in patients with traumatic brain injury (TBI) (Cox 2006; Cox 2012; SanBio 2016). However, their mesodermal potency poses a risk for unwanted differentiation after transplantation (Grigoriadis et al. 2011).

An alternative could be the use of autologous adult neural progenitor stem cells that can be isolated from easily accessible tissues in the adult body, such as periodontal ligament surrounding the teeth, soft palate, inferior turbinate, or hair follicles (Fernandes et al. 2004; Hauser et al. 2012; Sieber-Blum and Grim 2004; Techawattanawisal et al. 2007). These stem cells derive from a rich source of multipotent stem cells called the neural crest. It has been shown that neural crest-derived stem cells (NCSCs), harvested from adult hair follicles and implanted in a lesioned spinal cord, resulted in the production of cells that fulfill most criteria for a genuine neuronal differentiation (Hu et al. 2010). For cell-based therapy, the use of NCSCs from the hair follicle bulge (or hair follicle bulge-derived stem cells, HFBSCs) has several advantages above using other stem cell types, such as embryonic stem cells and neural stem cells. These advantages are (i) they are abundant and easily accessible and only minimally invasive surgery is necessary to harvest them; (ii) they are suitable candidates for autologous transplantation, which would avoid rejection of the transplant and graft-versus-host disease due to immunomodulation (Paus et al. 2005); and (iii) there is no evidence for tumor formation (Sieber-Blum et al. 2004). Besides, the hair follicle is an immune-privileged site indicating HFBSC tolerance in xenogeneic and allogeneic transplantations (Paus et al. 2005). In previous studies, we were able to isolate HFBSCs and investigate their proliferation rate, doubling time and cellular senescence as well as their capability to adapt a neuronal phenotype (Gho et al. 2015; Schomann et al. 2017; Schomann et al. 2016).

However, successful translation of stem cell-based therapies in the clinics will require robust preclinical testing and validation, focusing on consensual definitions of terms and the elucidation of the cells’ exact mechanism of action (Jendelova et al. 2016). An integral part of this is the requirement to monitor the extension of the TBI and response to treatment in a non-invasive manner, which could be facilitated by the application of multimodal imaging tools.

In order to regenerate neural tissue, transplanted stem cells have to survive, differentiate into neurons and/or glial cells and seek appropriate neuronal connections in order to achieve functional neuronal repair. Visualization of the migration and the fate of the transplanted stem cells in the injured brain in living animals could provide direct monitoring of the cells’ behavior after transplantation.

The aim of this study is to combine multimodality imaging technologies, such as optical imaging by means of bioluminescence imaging (BLI) and magnetic resonance imaging (MRI), to investigate in vivo the behavior of transplanted HFBSCs cells in a mouse model of TBI (Mezzanotte et al. 2011; Mezzanotte et al. 2017). The ultimate goal is to achieve a better understanding of the mechanism of recovery (e.g., cell replacement, support of the microenvironment, or a combination of both). This may ultimately contribute to a better strategy towards the promotion of neural tissue regeneration.

For this purpose, HFBSCs were transfected with a lentiviral vector containing a construct that is composed of a promoter and genes coding for both codon-optimized firefly luciferase (Luc2) and copepod green fluorescent protein (copGFP), in which the latter demonstrates a high fluorescence quantum yield and is more stable at a wide range of temperatures. Both genes are coupled via a T2A-like sequence, which mediates co-translational cleavage and, hence, results in bicistronic expression of copGFP and Luc2 (Abbas et al. 2000). The promoter is either the constitutively active elongation factor 1α (EF1α) or the promoter of doublecortin (DCX), which is a neuronal migration protein and early neuronal marker (Tennstaedt et al. 2015).

Additionally, transduced cells were loaded with ferumoxytol, which is a superparamagnetic iron oxide (SPIO) nanoparticle, in order to confirm their anatomical location by means of MRI in vivo (Pirko et al. 2005). Ferumoxytol is an FDA-approved drug for treatment of iron deficiency anemia and has been successfully used to image MSCs or macrophages loaded with heparin-protamine-ferumoxytol (HPF) complexes in preclinical studies (Khurana et al. 2012; Thu et al. 2012).

## Material and methods

### Cell culture

Hair follicles were dissected out from the whisker pads of healthy adult (> 23 days) male and female surplus mice (strain C57Bl/6), as previously described (El Seady et al. 2008; Gho et al. 2016). Isolation of HFBSCs from the bulge region of the hair follicle was described elsewhere (Gho et al. 2016; Schomann et al. 2016; Sieber-Blum et al. 2004).

Selected HFBSCs were cultured on poly-D-lysine-coated (PDL; Sigma-Aldrich, St. Louis, MO, USA; 0.01 mg/ml in distilled water) cell culture-treated 12-well plates (TPP Techno Plastic Products AG, Trasadingen, Austria) in basic growth medium (BGM) and maintained in a humidified incubator at 37°C and 5% CO_2_. BGM, modified from Nguyen et al. (Nguyen et al. 2013), consists of DMEM/Ham’s F-12 1:1 (Biochrom AG, Berlin, Germany), 1% GlutaMax™ (100×; Gibco, Bleiswijk, the Netherlands) and 1% antibiotic/antimycotic solution (100×; Sigma-Aldrich) supplemented with 10% fetal bovine serum (FBS; Gibco), 2% B-27® supplement without vitamin A (50×; Gibco), 1% N-2 MAX media supplement (100×; R&D Systems™, Minneapolis, MN, USA), recombinant human basic fibroblast growth factor (20 ng/ml; R&D Systems) and recombinant human epidermal growth factor (20 ng/ml; R&D Systems). The cultures were passaged, as previously described, after 60–70% confluence was reached (Gho et al. 2016; Schomann et al. 2016). HFBSCs were frozen in 10% DMSO in FBS and stored at − 80°C until use. After thawing, HFBSCs were cultured in BGM on PDL-coated dishes (TPP Techno Plastic Products AG) and used for subsequent experiments.

### Lentiviral vector production and transduction of HFBSCs

HFBSCs were transduced with a third-generation lentiviral vector containing the sequences for Luc2 and copGFP. Details on these reporter molecules as well as their cloning and recombination procedures have been reported previously (Boehm-Sturm et al. 2014; Löw et al. 2010; Mezzanotte et al. 2013). In brief, lentivirus particles were generated by means of transfection of HEK293 cells with packaging plasmids and the plasmid pCDH-EF1-Luc2-T2A-copGFP or pCDH-DCX-Luc2-T2A-copGFP (Schomann et al. 2016). Virus was quantified by antigen-capture ELISA, measuring HIV p24 levels (ZeptoMetrix Corporation, NY, USA). For transduction, HFBSCs were resuspended in BGM. Pseudoviral particles containing the copGFP-Luc2 constructs, using 40 ng virus per 1 × 10^5^, were added to the cells. Transduced HFBSCs were stored at − 80°C until use.

Lentiviral vector production and stem cell transduction were performed under the appropriate biosafety level conditions (ML-II) in accordance with the National Biosafety Guidelines and the Regulations for Research on Genetically Modified Organisms. Procedures and protocols were reviewed and approved by the LUMC Biosafety Committee (GMO permit 00-026).

### Fluorescence microscopy

Transduced HFBSCs containing the copGFP-Luc2 construct were plated and allowed to attach in PDL-coated 12-well cell culture plates. Expression of copGFP was observed using an Olympus IX70 epi-illumination fluorescence microscope (FITC filter settings) with a Leica DFC340 FX digital color camera (Leica Camera AG, Wetzlar, Germany). Images were acquired and digitally stored using Leica Application Suite Advanced Fluorescence (LAS-AF) version 1.9 software.

### In vitro differentiation

Expression of copGFP under regulation of the DCX promoter was investigated by differentiating pCDH-DCX-Luc2-T2A-copGFP-transduced HFBSCs, according to a previously established neural differentiation protocol (Gho et al. 2016; Schomann et al. 2016). pCDH-EF1α-Luc2-T2A-copGFP-transduced HFBSCs served as controls. Briefly, 2.5 × 10^5^ cells were seeded via the side into PDL-coated wells containing PDL-coated cover glasses (Thermo Scientific, Waltham, MA, USA) in a total volume of 500 μl BGM. Differentiation was induced by replacing 250 μl medium with 300 μl induction medium (IM). IM consists of DMEM/Ham’s F-12 1:1 supplemented with 1.5 mM cAMP (Sigma-Aldrich), 1% GlutaMax (Life Technologies), 10 ng/ml NGF, 10 ng/ml GDNF, 10 ng/ml BDNF (all from R&D Systems) and 2% B27 with vitamin A (Life Technologies). Subsequently, cultures were allowed to differentiate for at least 60 h without disturbance, followed by an additional substitution of 250 μl medium with 300 μl IM.

### Loading of HFBSCs with HPF complexes

HFBSCs containing the copGFP-Luc2 construct were loaded with HPF complexes, according to the procedure of Thu et al. (Thu et al. 2012). An amount of 4 × 10^6^ cells were re-suspended in serum-free BGM containing 2 IU/ml sodium heparin (LEO Pharma, Amsterdam, the Netherlands), 60 μg/ml protamine hydrochloride (MEDA Pharma BV, Amstelveen, the Netherlands) and 50 μg/ml ferumoxytol (Rienso®, Takeda Pharma A/S, Roskilde, Denmark), which was then followed by incubation at 37°C for 2 h. An equal amount of BGM containing 20% FBS was added, upon which the cells were transferred to PDL-coated dishes and incubated in a humidified incubator with 5% CO_2_ at 37°C. After 24 h, the cells were firstly washed with phosphate-buffered saline (PBS) and then with PBS containing heparin (10 IU/ml). The cells were passaged by adding pre-warmed (37°C) balanced salt solution containing 0.05% trypsin and 0.02% EDTA.4Na (Gibco Life Technologies) to the culture dish, before being incubated for 2 min. Cells were collected and re-suspended in PBS at a concentration of 5 × 10^4^ cells/μl and stored at 4°C until transplantation.

### Detection of ferumoxytol

To visualize HPF complexes that were endocytosed by HFBSCs, cells were fixed with pre-warmed 1% formaldehyde in PBS directly after incubation. Fixed ferumoxytol-loaded HFBSCs and mouse brain cryosections were pre-treated with 3% H_2_O_2_ in methanol for 30 min to inhibit endogenous peroxidase activity. This was followed by washing in distilled water for 30 min. Iron oxide-containing ferumoxytol was visualized using Perls’ Prussian blue method, followed by 3,3′-diaminobenzidine (DAB) intensification (Meguro et al. 2007). Cells were then incubated in 1% potassium ferrocyanide (K_4_Fe(CN)_6_.3H_2_O) with 1% HCl in distilled water for 30 min, which was then followed by several washes in distilled water (3 × 10 min). Next, the specimens were incubated in the dark for 10 min in a solution containing 0.1% 3,3′-diaminobenzidine, 4% HCl and 0.03% H_2_O_2_ in PBS. This was followed by 3 washes in distilled water (5 min each) to stop the reaction. Specimens were subsequently mounted in Roti®-Mount FluorCare mounting medium (Carl Roth GmbH + Co. KG, Karlsruhe, Germany) and examined with a Leica DM5500B microscope with a Leica DFC 450C color camera. Digital images were acquired and stored using Leica Application Suite (LAS V4.5) software.

### Animals and TBI

Animal care and handling were in accordance with the guidelines and regulations as stipulated by the Dutch Experiments on Animals Act (WoD) and the European Directive on the Protection of Animals Used for Scientific Purposes (2010/63/EU). All applicable institutional and national guidelines for the care and use of animals were followed.

Healthy 8-week-old female CD1-nude mice (*n* = 10; Charles River, Chatillon-sur-Chalaronne, France) were used for the transplantation experiments. Mice were housed in the Animal Care Facility of Leiden University Medical Center (LUMC, the Netherlands) under standard housing conditions (group cages with enriched environment, food and water *ad libitum*; diurnal light cycle (12 h light, 12 h dark), temperature 21°C; humidity 60%). The use of the animals was approved by the Animal Experiments Committee of the Leiden University Medical Center (DEC permits 10065, 11198/3, and 13024/1).

Anesthesia was induced for all experiments with 4% isoflurane in air (Teva Pharmachemie BV, Haarlem, the Netherlands) and mice were kept under anesthesia with 1.5% isoflurane in air. TBI was induced using a liquid nitrogen pre-cooled copper conical cylinder with a 3-mm diameter tip. The cylinder was applied to the head of each mouse approximately 3 mm left of the bregma for 40 s, so as to induce traumatic brain damage as previously described (Smith et al. 2012).

Two days after induction of the TBI, mice were anesthetized with 2% isoflurane in air for transplantation of transduced, ferumoxytol-loaded HFBSCs. A motorized, computer-controlled stereotaxic instrument (Neurostar, Tübingen, Germany) with mouse brain atlas integration and real-time visualization of the injection site in the atlas space was used for in vivo injections of HFBSCs into the mouse brain. The anesthetized mouse was placed into the stereotaxic instrument that fixes the skull with ear bars and a clamp system that tightens against the jawbone and the palate. The coordinates for injection were X (− 2), Y (2) and Z (1) relative to the anterior bregma. The needle was navigated by a motorized stereotactic frame utilizing StereoDrive software. Each injection was carried out within a standardized time frame, i.e., 1 min injection time and 2 min deposition rest before needle retraction, to prevent potential variations in the effect of shearing forces. A total volume of 2 μl containing 2 × 10^5^ HFBSCs was stereotactically transplanted into the cerebral cortex of the animals.

### Bioluminescence imaging

Observation of survival (pCDH-EF1α-Luc2-T2A-copGFP) and differentiation (pCDH-DCX-Luc2-T2A-copGFP) of transplanted HFBSCs were achieved using BLI at 2, 14, 33 and 49 days after transplantation. Prior to imaging, mice received an intraperitoneal injection of D-luciferin potassium salt (Synchem UG & Co. KG, Felsberg, Germany). Anesthesia was induced with 4% isoflurane in air and mice were kept under anesthesia with 1.5% isoflurane in air. Images were acquired 15 min after injection of D-luciferin (150 mg/kg) using 30-s exposure, open filter, field of view C (default setting), f/stop = 1 and medium binning for all bioluminescence measurements. All imaging measurements were performed with the IVIS® Spectrum multimodal imaging system (Caliper Life Sciences, Hopkinton, MA, USA), which combines laser scan surface topography with BLI, with the stage warmed to 37°C. Image acquisition and analysis were performed with Living Image version 4.2.1 software (Caliper Life Sciences).

### Magnetic resonance imaging

In addition to BLI, ferumoxytol-loaded HFBSCs were imaged after their transplantation into the mouse brain with TBI using MRI to investigate the exact location of the stem cells at 1 and 48 days after transplantation. MRI was performed with a 7-T Bruker PharmaScan® 70/16 (Bruker Biospin, Ettlingen, Germany) equipped with a BGA-9S 300 mT/m gradient system and a conventional 23-mm birdcage transmit-and-receive radio-frequency (RF) coil (Bruker Biospin). Mice were initially anesthetized with 4% isoflurane in air and kept under anesthesia with 1.5% isoflurane in air throughout the imaging procedure. Mice were placed in the RF coil, fixed in the bed with ear bars and kept warm using a water-heated pad with thermo-coupling to control mouse temperature. Respiration rate and temperature were measured continuously. After an initial localization scan, T2*-weighted three-dimensional fast low-angle shot (FLASH) sequences were used to visualize the mouse. Optimal sequence parameters were as follows: repetition time (TR), 100 ms; effective echo time (TE), 13 ms; imaging matrix size, 128 × 128 × 64; final voxel resolution of 219 × 219 × 250 μm; and a FOV of 28 × 28 × 16 mm. Data acquisition, image reconstruction and visualization were achieved with Paravision® 6.0.1 software (Bruker Biospin, Ettlingen, Germany). Images were processed using ImageJ image analysis software (https://imagej.nih.gov/ij/plugins/cell-counter.html; version 1.47; US National Institutes of Health, Bethesda, MD, USA).

### Immunohistochemistry

After 58 days, the anesthetized animals were fixed by means of intracardial perfusion with 4% formaldehyde in PBS. After decapitation, the brains were removed and stored in 1% formaldehyde in PBS at 4°C until further processing. Next, specimens were embedded in Tissue-Tek® O.C.T.™ (Sakura Finetek Europe B.V., Alphen aan den Rijn, the Netherlands) compound and frozen for cryosectioning at − 20°C. Frozen mouse brains were cut with the Microtome Cryostat HM 500 OM (MICROM International GmbH, Walldorf, Germany), 10 μm brain sections were transferred onto KP Plus slides (Klinipath B.V., Duiven, the Netherlands) and the slides were stored at − 20°C.

For immunohistochemistry, sections were processed as previously described (Gho et al. 2016). This procedure was also followed for the cultured cells. Primary and secondary antibodies used in this study are listed in Table 1. For all stainings, proper positive and negative controls were used. Adult human dermal fibroblasts (HDFa) from ScienCell (catalog number: 2320; ScienCell, Carlsbad, CA, USA) were obtained from the donors after signed informed consent and in compliance with local, state and federal laws and regulations. Nuclei were counterstained with 1:1000 DAPI (Invitrogen) in PBS for 15 min. The specimens were mounted in a drop of Roti®-Mount FluorCare (Carl Rothi GmbH+Co. KG, Karlsruhe, Germany). All specimens were examined with a Leica DM5500 B fluorescence microscope (filter settings: TXR, Cy7, FITC, and DAPI), equipped with a Leica DFC365 FX digital camera. Digital images were acquired and stored using Leica Application Suite X (LAS X) software.

**Table 1:** Antibodies

Type	Antibody	Host	Clon	Company	Cat. No.	Localization	Dilution	Control
Primary	CopGFP	Rabbit	polyclonal	Evrogen	AB513	Cytoplasm	1:200	Luc2-copGFP transduced cells
Primary	Doublecortin (DCX)	Rabbit	polyclonal	Abcam	Ab18723	Cytoplasm	1:200	C17.2, Brain (Mouse) tissue lysate - normal tissue, 0 days old
Primary	Fibronectin	Rabbit	polyclonal	Sigma-Aldrich	F3648	Extracellular matrix glycoprotein	1:400	
Primary	GFAP	Rabbit	polyclonal	DAKO	Z0334	Cytoplasm	1:500	RT4-D6PT2
Primary	Ki-67	Rabbit	polyclonal	Abcam	ab15580	Nuclear	1:100	Human dermal fibroblasts
Primary	Laminin	Rabbit	polyclonal	Dako	Z009701	Extracellular matrix	1:200	RT4-D6P2T, MelbA, HFBSCs
Primary	Luc2	Mouse	monoclonal	DSHB	DSHB-LUC-2	Cytoplasm	25:100	Luc2-copGFP transduced cells
Primary	Nestin 4D11	Mouse	monoclonal	Biosensis	M-1385-100	Cytoplasm	1:300	C17.2 (NSCs), M14, RT4-D6P2T
Primary	NF-Pan	Mouse	monoclonal	EMD Millipore	NE1017	Cytoskeleton	1:1000	Mouse brain slices
Secondary	Alexa Fluor™ 555	Goat	polyclonal	BioLegend	405,324		1:200	
Secondary	Alexa Fluor™ 750	Goat	polyclonal	Abcam	ab175733		1:200	

### Statistical analysis

Statistical analyses of the data were performed using GraphPad Prism 6.02 and SPSS Statistics version 20.0.0.1 software (IBM Corporation, Armonk, NY, USA). A multiple *t-*test was applied to the cytokine assay data and data were expressed as mean ± standard error of the mean (SEM). A one-way ANOVA with Bonferroni’s multiple comparison test and 95% confidence interval was applied to the BLI data and data were expressed as mean ± SEM.

## Results

### In vitro differentiation of transduced HFBSCs

**Fig. 1:**
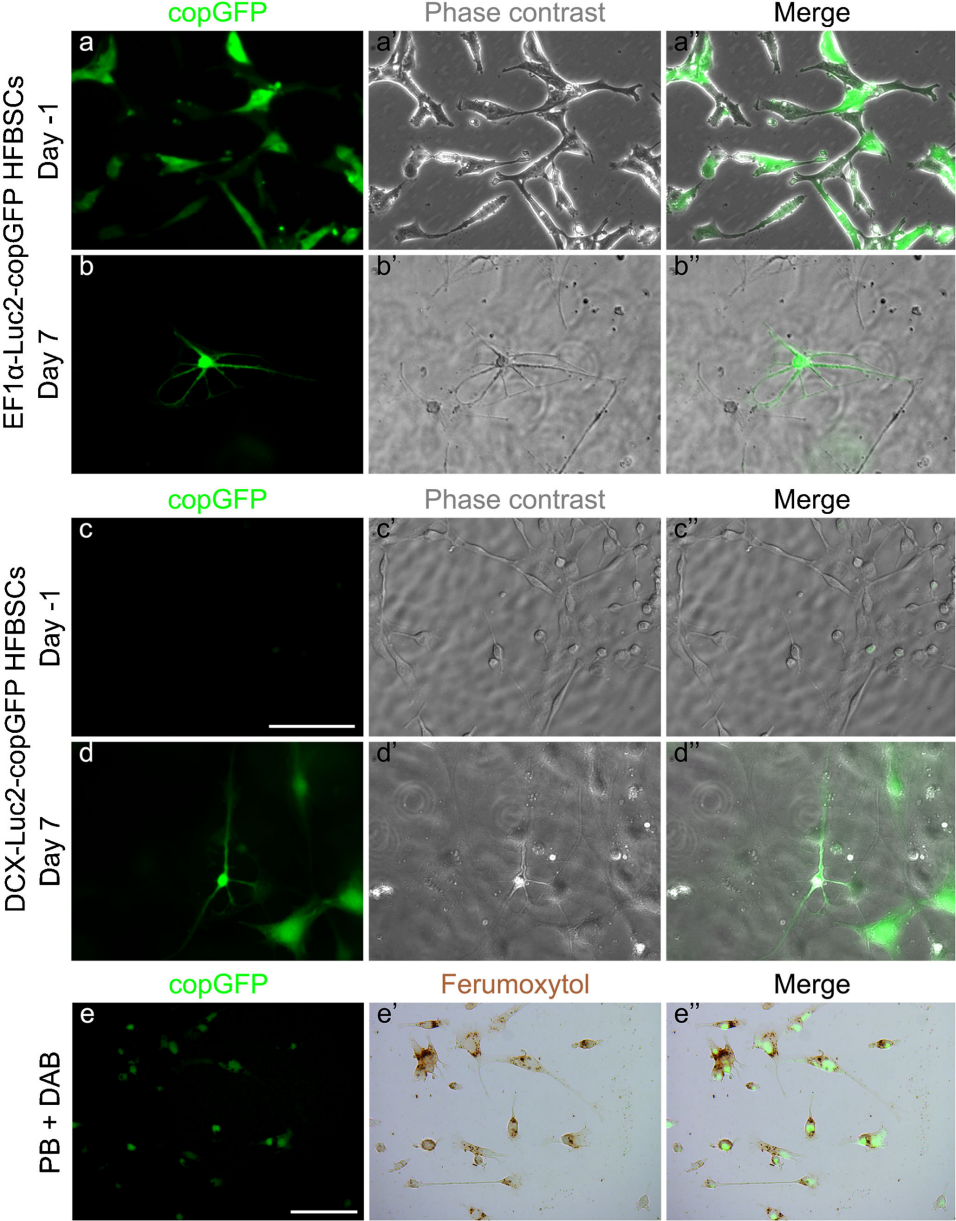
In vitro differentiation and loading with ferumoxytol of transduced HFBSCs. Prior to differentiation (day − 1) pCDH-EF1α-Luc2-T2A-copGFP-transduced cells exhibited a bright green-fluorescent signal of copGFP (a) and had normal morphologies (a″; phase contrast). The merged image (a‴) demonstrates that all cells were transduced with the reporter gene construct. Within 7 days, HFBSCs adapted neuronal morphologies (b; copGFP fluorescence and b′; Phase contrast). Scale bar is 100 μm. HFBSCs transduced with the pCDH-DCX-Luc2-T2A-copGFP construct did not express copGFP (or Luc2) prior to differentiation (day − 1) as indicated by the absence of a fluorescent signal (c). However, cells expressed copGFP under regulation of the DCX promoter as indicated by the green fluorescent signal 7 days after start of the differentiation (d). HFBSCs also adapted neuronal morphologies (d′; phase contrast). Scale bar is 100 μm. Perls’ Prussian blue (PB) staining + DAB intensification stained ferumoxytol within the cells as marked by a brown precipitate (e′). Faint copGFP fluorescence persisted through the staining process as can be observed in the fluorescence (e) and merged images (e″). Scale bar is 100 μm

HFBSCs, transduced with the pCDH-EF1α-Luc2-T2A-copGFP gene construct, constitutively express both Luc2 and copGFP at equimolar ratios. Prior to differentiation, the HFBSCs exhibited a bright green-fluorescent signal and normal morphologies (Fig. 1a–a″). The merged image demonstrates that all cells were successfully transduced with the reporter gene construct (Fig. 1a″). After induction of neuronal differentiation, HFBSCs adopted neuronal morphologies within 7 days as shown in the fluorescence and phase-contrast images (Fig. 1b–b″).

Under the regulation of the promoter for the neuronal migration protein DCX, HFBSCs that were transduced with the pCDH-DCX-Luc2-T2A-copGFP construct did not express copGFP (or Luc2) during standard cell culture as marked by the absence of any fluorescent signal (Fig. 1c–c″). However, after induction of neuronal differentiation, HFBSCs showed both neuronal morphologies and copGFP expression under regulation of the DCX promoter within 7 days (Fig. 1d–d″).

### Confirmation of ferumoxytol uptake by HFBSCs

To confirm that HFBSCs could take up the MRI contrast agent ferumoxytol, we performed Perls’ Prussian blue staining with DAB intensification on transduced cells loaded with HPF complexes. While the cells constitutively expressed copGFP (Fig. 1e), Perls’ Prussian Blue staining with DAB intensification revealed the presence of iron oxide within 92.2 ± 1.1% of the cells as marked by intracellular deposits of a brown precipitate (Fig. 1e–e″).

### Bioluminescence imaging of HFBSCs transduced with pCDH-EF1α- and pCDH-DCX-Luc2-T2A-copGFP in mice with TBI

**Fig. 2:**
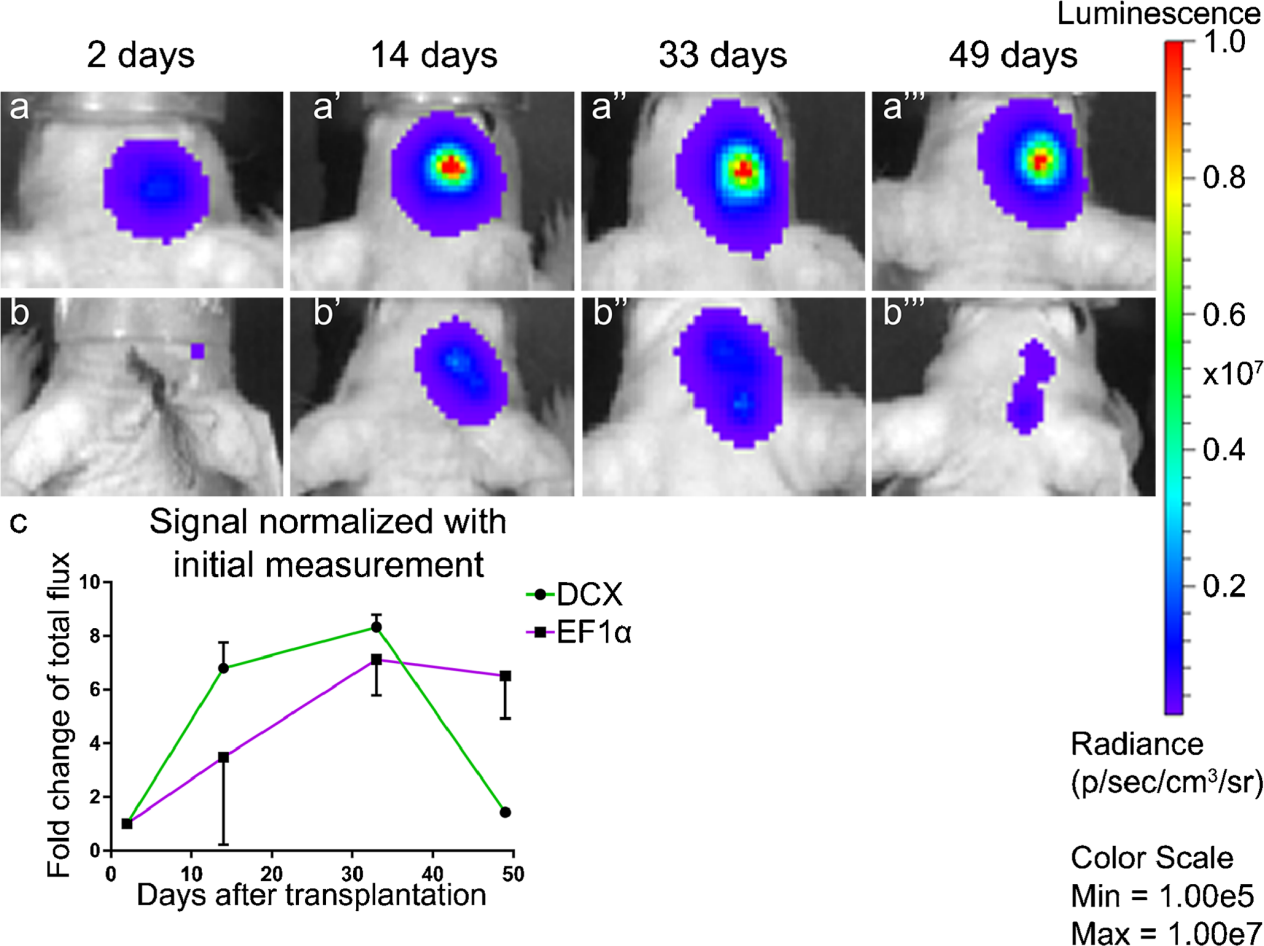
Observation of Luc2 activity in TBI mice in vivo. Representative overlays of pCDH-EF1α-Luc2-T2A-copGFP-transduced HFBSCs from 2 (a), 14 (a′), 33 (a″) and 49 days after transplantation (a‴). The bioluminescent signal increased with relative stability over the course of time. Representative overlays of HFBSCs transduced with pCDH-DCX-Luc2-T2A-copGFP over the same period of time. No bioluminescent signal was observed 2 days after transplantation (b). The signal increased between 14 days (b′) and 33 days (b″) but was almost undetectable after 49 days (b‴). Analysis of the bioluminescent signal was measured 2, 14, 33 and 49 days after transplantation (c). The bioluminescence data were normalized with the initial signal and measured 2 days after transplantation, which depicts the trend of the bioluminescent signal from HFBSCs over time. The bioluminescent signal of pCDH-EF1α-Luc2-T2A-copGFP-transduced HFBSCs increased steadily over the course of time, while the bioluminescence of pCDH-DCX-Luc2-T2A-copGFP-transduced HFBSCs decreased after 33 days and was almost undetectable at 49 days

After transplantation, the bioluminescent signal of pCDH-EF1α-Luc2-T2A-copGFP-transduced HFBSCs showed a slow and stable increase over a period of 49 days (Fig. 2a–a‴). Cells transduced with the pCDH-DCX-Luc2-T2A-copGFP gene construct revealed a faint or absent bioluminescence, 2 days after transplantation (Fig. 2b′–b‴). However, representative overlays show that a distinct bioluminescent signal emanated from the injection site within 14 days (Fig. 2b′) and peaked 33 days after transplantation (Fig. 2b″). Interestingly, the bioluminescent signal dropped below initial levels and almost vanished 49 days after transplantation of transduced HFBSCs (Fig. 2b‴). A strong bioluminescent signal was obtained from transplanted HFBSCs containing the pCDH-EF1α-Luc2-T2A-copGFP construct, while it was not significantly different between the days of the measurements (one-way ANOVA, *p* = 0.14) (Fig. 2c). The bioluminescent signal from pCDH-DCX-Luc2-T2A-copGFP-transduced HFBSCs initially increased over the period of 33 days and thereafter steadily decreased, which was statistically significant (one-way ANOVA, *p* = 0.048; Fig. 2c).

### MRI of TBI mice transplanted with ferumoxytol-loaded HFBSCs

**Fig. 3:**
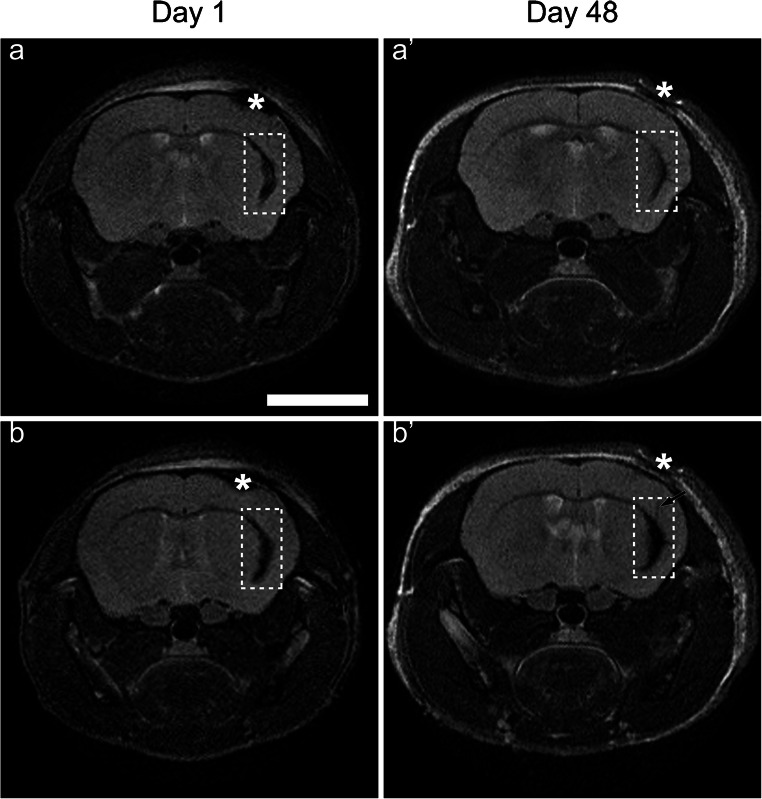
MRI of ferumoxytol-loaded HFBSCs in TBI mice at different Bregma levels in vivo. Different sources of T2*-weighted contrast are illustrated in areas close to the TBI or more remotely. The upper panel shows representative scans of the region containing the TBI lesion 1 day (a) and 48 days after transplantation (a′). The scan at day 1 revealed a hypointense area containing clotted iron-containing erythrocytes (asterisk), which vanished over the course of time. Dotted box: injected cells. The lower panel shows representative scans of the transplanted ferumoxytol-loaded transduced HFBSCs over the same course of time (b, b′). The dotted box at day 1 after transplantation shows the location of ferumoxytol-containing cells. Asterisk: clotted erythrocytes. The hypointense signal of ferumoxytol could also be imaged at the transplantation site at least 48 days after transplantation. Scale bar is 200 μm

In order to enable visualization of the cells by means of MRI, HFBSCs were loaded with ferumoxytol prior to transplantation. The TBI lesion, which was induced 2 days before transplantation of the HFBSCs, was clearly visible on the MRI due to iron-rich, clotted erythrocytes (Fig. 3a, asterisks). In addition to the lesion, the transplanted ferumoxytol-loaded HFBSCs (Fig. 3a, dotted boxes) were also visible. Over the course of 8 days, the hypointense signal from the iron-containing erythrocytes at the site of the TBI lesion completely disappeared (data not shown; cf., Fig. 3a′, day 48). Ferumoxytol-loaded HFBSCs were visible as a hypointense area within the mouse brain 1 day after injection (Fig. 3b, day 1, dotted boxes). Subsequent MRI of mice revealed that a hypointense area from ferumoxytol persisted within the brain for at least 48 days and showed migration of the hypointense signal towards the site of TBI (Fig. 3b′, day 48, arrow).

### Histochemical staining

**Fig. 4:**
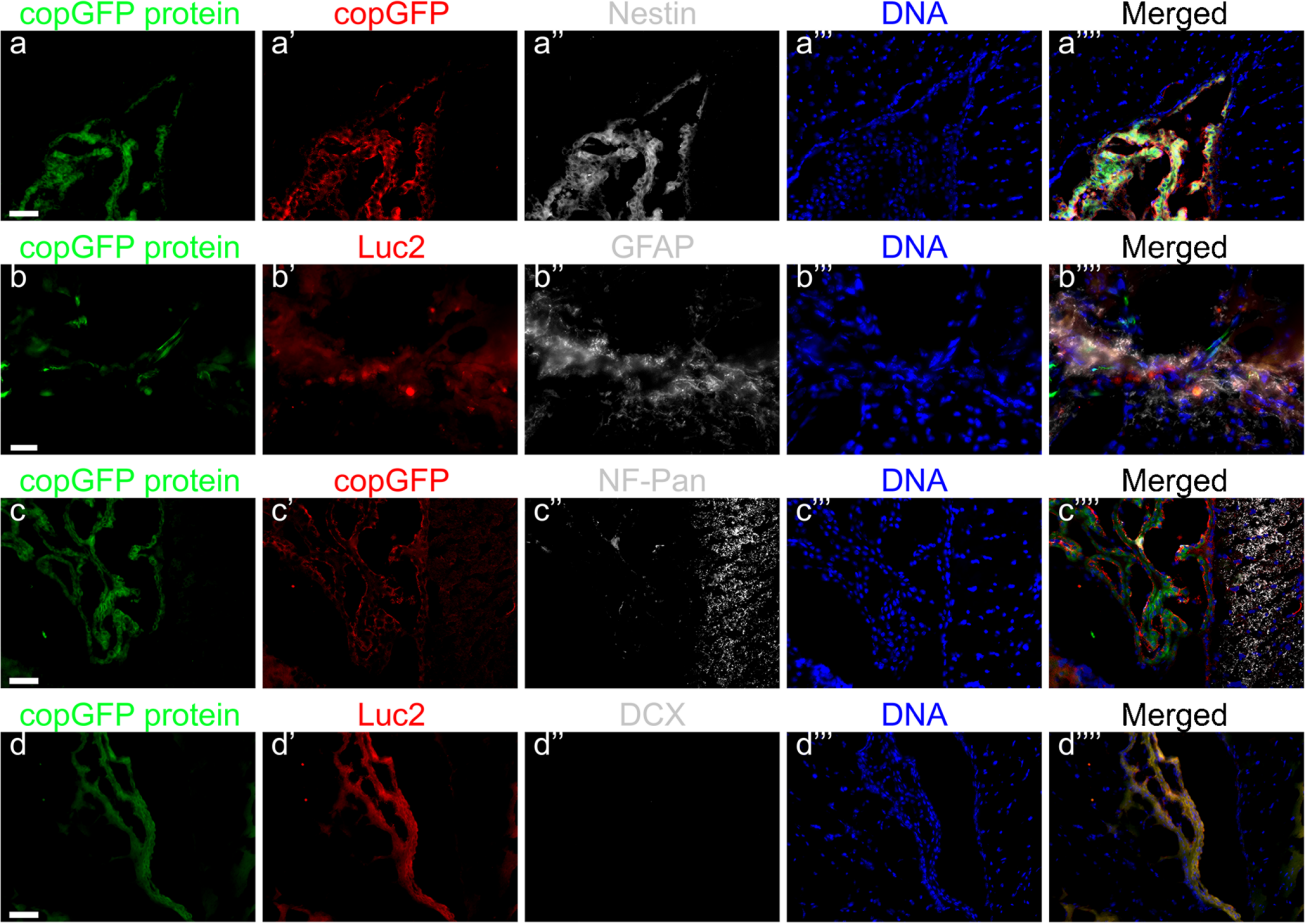
Immunohistochemical staining of HFBSCs constitutively expressing Luc2 and copGFP. Sections of mouse brains containing transduced HFBSCs exhibited native green fluorescence emitted by copGFP after fixation, sectioning and staining of the sections (a, b, c, d; green). Sections containing copGFP-expressing HFBSCs were stained for either copGFP (a′ and c′) or Luc2 (b′ and d′). CopGFP-expressing HFBSCs stained for copGFP (a′; red) and the neural progenitor cell marker nestin (a″; gray). The merged image (a′′′′) shows colocalization of copGFP (a′), nestin (a″) and DNA (a‴) of copGFP-expressing HFBSCs in the mouse brain. HFBSCs, which expressed copGFP (b; green), also stained for Luc2 (b′; red). GFAP (b″; gray) stained in the mouse brain but is absent in HFBSCs (b‴; green/red). Transplanted HFBSCs (c/c′; green/red) stained for NF-Pan (c″; gray). None of the copGFP-expressing HFBSCs (d/d′; green/red) stained for DCX (d″). Scale bar = 50 μm

**Fig. 5:**
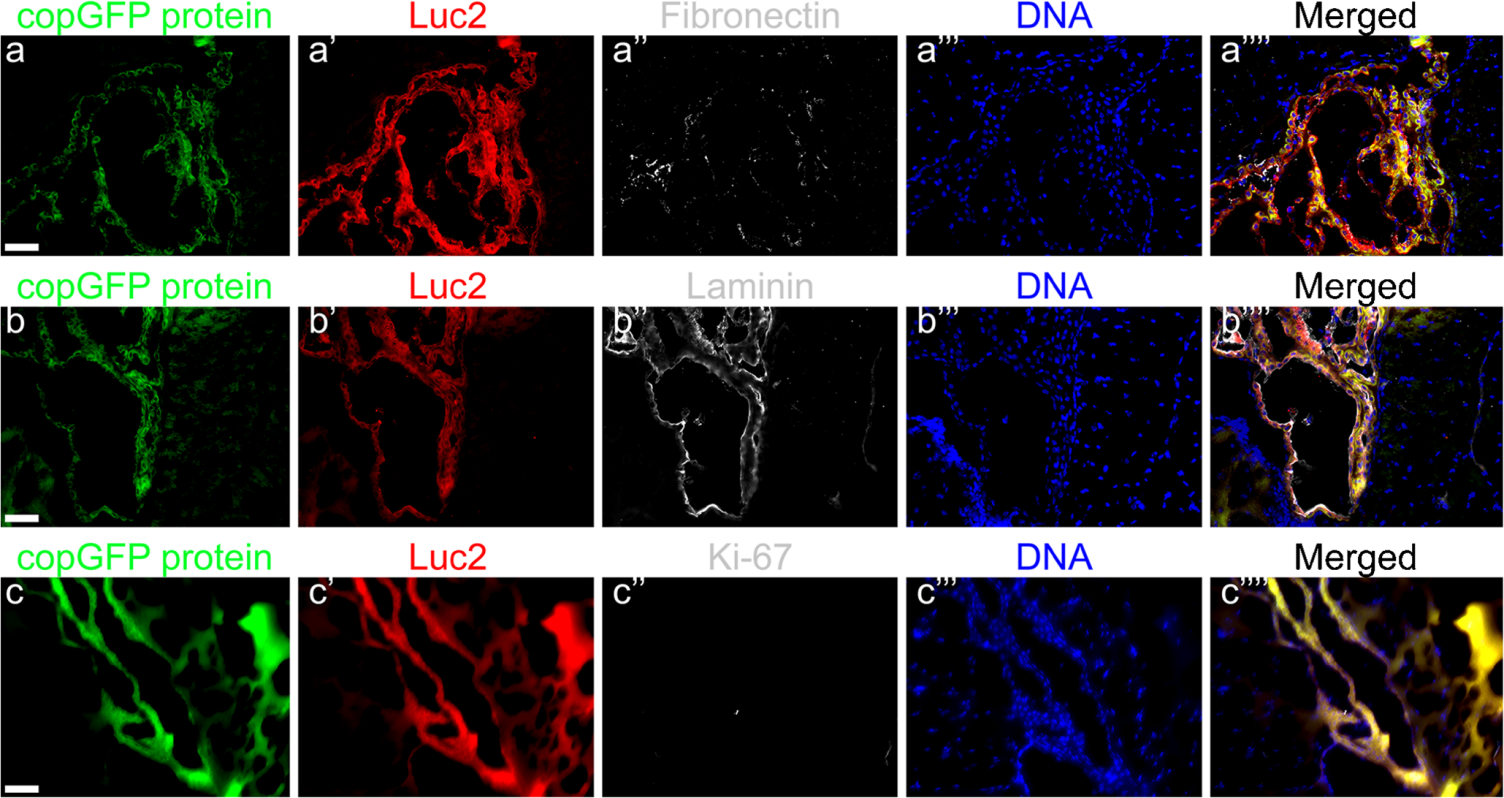
Immunohistochemical staining for extracellular matrix and proliferating cells. CopGFP-expressing HFBSCs in mouse brain sections exhibited native fluorescence (a, b, and c; copGFP protein; green). Additionally, sections were stained for Luc2 (a′, b′, and c′; red). The surrounding area of some HFBSCs stained for fibronectin (a″; gray). The vicinity of transplanted HFBSCs stained for laminin (b″; gray). Staining for Ki-67 (c″; gray) was negative in sections containing HFBSCs (green/red). Scale bar = 50 μm

**Fig. 6:**
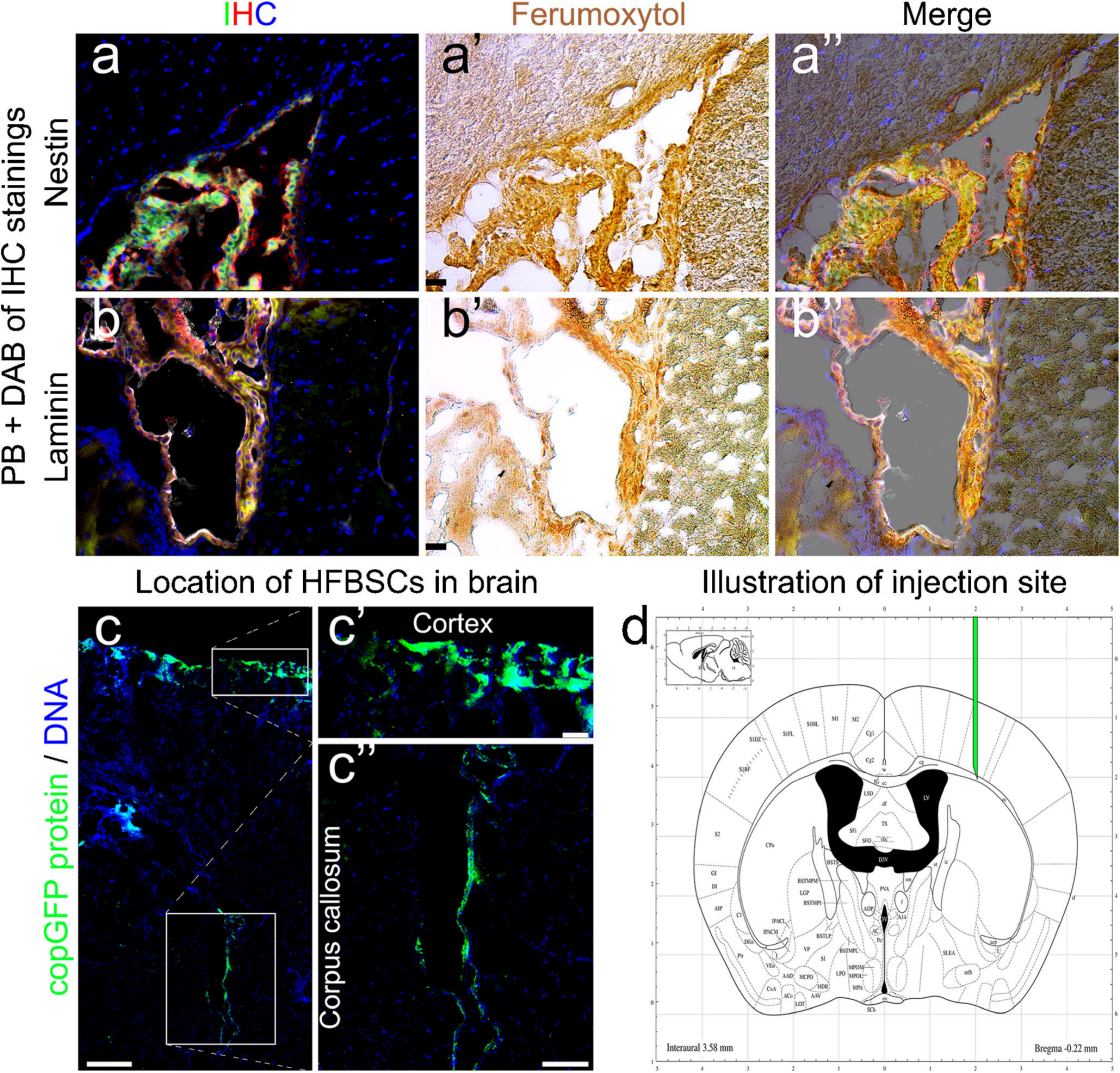
Perls’ Prussian blue staining with DAB intensification of mouse brain sections. Immunohistochemically stained sections from Fig. 4(a–a′′′′) and Fig. 5(b–b′′′′) (IHC) were also stained with Perls’ Prussian blue with DAB intensification. This showed that HFBSCs stain for ferumoxytol 58 days after transplantation in TBI mice (a′ and b′; ferumoxytol). The merged image of the ferumoxytol staining and the corresponding immunofluorescence image reveals colocalization of Fe^3+^ deposits and a fluorescent signal (a″ and b″; merged). Scale bar is 25 μm. The native fluorescence of the copGFP, which persists also after the preparation of the brain sections, indicates the location of the HFBSCs within the mouse brain (c; scale bar is 250 μm), i.e., the cortex (c′; scale bar is 50 μm) and the corpus callosum (c″; scale bar is 100 μm; enlarged images of boxes). Illustration of the injection site of HFBSCs in the mouse brain (d; adapted from the Neurostar Robotic Stereotactic Software)

The stability of copGFP protein enabled visualization of the fluorescent protein in cryosections of mouse brains without additional immunohistological staining for copGFP (Fig. 4a–d and Fig. 5a–c, copGFP protein). However, we performed an additional immunostaining of the cryosections for copGFP (Figs. 4a′ and c′,) or Luc2 (Figs. 4b′, d′, and 5a′–c′) to confirm the presence of pCDH-EF1α-Luc2-T2A-copGFP-transduced HFBSCs. Furthermore, all cells that exhibited green fluorescence from copGFP also showed staining for the neural progenitor cell marker nestin (Fig. 4a″). A marker for astrocytes in the central nervous system, GFAP, showed staining in the mouse brain but not in HFBSCs (Fig. 4b″). The depicted GFAP signal originates from glial cells surrounding the copGFP expressing HFBSCs. Staining for the neural marker neurofilament with NF-Pan was faint and restricted to individual HFBSCs but colocalized with copGFP (Fig. 4c″). Staining of the transduced HFBSCs for DCX was negative (Fig. 4d″). In addition, the surrounding area of some green fluorescent HFBSCs also stained for fibronectin (Fig. 5a″) and laminin (Fig. 5b″). This indicates secretion of these extracellular matrix proteins by the injected cells. Staining for the proliferation marker Ki-67 was negative in transplanted HFBSCs (Fig. 5c″) but positive in HFBSCs in vitro (Table 2). Positive and negative controls showed the reliability of the staining performed (data not shown). Table 2 compares the immunostaining of cultured HFBSCs and the cryosections of TBI mouse brains. Perls’ Prussian blue staining with DAB intensification of cryosections, which were immunohistochemically stained for nestin (Fig. 6a) and laminin (Fig. 6b), showed that HFBSCs (which also expressed copGFP and stained for copGFP or Luc2) stained for ferumoxytol after 58 days in vivo (Fig. 6a′ and b′). However, iron staining was also positive in the tissue surrounding the graft, which may indicate uptake of iron by other cells, e.g., macrophages, (Fig. 6a″ and b″, merge). Reconstruction of brain sections guided by fluorescence of copGFP (Fig. 6c) reconfirmed that, after transplantation, HFBSCs were present in the superficial cortex (where TBI was imaged by means of MRI) as well as in the deep cortex (Fig. 6c′) and corpus callosum (Fig. 6c″) next to the injection site as depicted in Fig. 6d.

**Table 2:** Overview of staining pattern

	Antibody	Cultured HFBSCs	Transplanted HFBSCs	Specification
HFBSCs	copGFP	Positive	Positive	Transduced HFBSCs expressing copGFP
	Luc2	Positive	Positive	Transduced HFBSCs expressing Luc2
Neural Crest	Nestin	Positive	Positive	Neural crest cells and neuronal progenitors
Neuron	DCX	Negative	Weakly positive	Early neuronal development
	NF-Pan	Weakly positive	Positive	Neurons
Glial Cell	GFAP	Negative	Negative	Glial cells in the peripheral and central nervous system
ECM	Fibronectin	Negative	Positive	Cell adhesion, growth, migration, differentiation, neuron protection
	Laminin	Positive	Positive	Cell attachment, stimulates neuronal differentiation, promotion of tissue survival
Other	Ki-67	Positive	Negative	Proliferating cells

## Discussion

We were able to show for the first time that HFBSCs can survive and differentiate towards a neuronal cell lineage after transplantation of these cells into the mouse brain by applying in vivo multimodal imaging, i.e., BLI and MRI.

We established that HFBSCs tolerate genetic manipulation and loading with nanoparticles in vitro in a previous study (Schomann et al. 2016). For the present study, we also loaded the HFBSCs with the (ultrasmall) SPIO nanoparticle ferumoxytol, which has proven effective for in vivo tracking of stem cells with high sensitivity by means of MRI (Gutova et al. 2013; Khurana et al. 2013; Pirko et al. 2005; Schieda 2013). We showed that HFBSCs take up HPF complexes using the Perls’ Prussian blue method and DAB intensification, which stains Fe^3+^ deposits within the cells.

In mice with TBI, the bioluminescent signal of HFBSCs transduced with pCDH-EF1α-Luc2-T2A-copGFP remained high over time, while bioluminescence of pCDH-DCX-Luc2-T2A-copGFP-transduced HFBSCs was initially low. It increased over a period of at least 33 days and completely vanished afterwards, which indicated that BLI can be used in tracking early differentiation in vivo. Tennstaedt et al. reported the same bioluminescence pattern of human neural stem cells transduced with Luc2 under regulation of the DCX promoter in their in vivo study (Tennstaedt et al. 2015). Our discovery that the bioluminescent signal vanished within 49 days after injection was also in line with the work of Ladewig et al., who demonstrated decreased DCX expression during cell maturation, which is likely due to the fact that DCX is a marker expressed early on in neural differentiation (Ladewig et al. 2008). In addition, we could rule out that the decrease in bioluminescent signal was due to cell death since the signal from pCDH-EF1α-Luc2-T2A-copGFP-transduced HFBSCs, which constitutively express Luc2, remained constant over the same period.

We checked for hypointense regions in the mouse brain by means of MRI at 1 day after transplantation of ferumoxytol-loaded transduced HFBSCs and again 48 days after transplantation. Bryant et al. recently demonstrated that formation of FHP complexes (ferumoxytol is the base component and heparin and protamine are added to form the complexes) improved the MRI contrast compared to HPF complexes (ferumoxytol is added as a last component) (Bryant Jr. et al. 2017). However, in our case, HFBSCs showed a high uptake of HPF complexes and could be clearly detected after transplantation next to the TBI lesion (cortical region) in nude mice at both time points. One day after transplantation, the MRI showed hypointense spots in the cortex area, indicating the presence of ferumoxytol. MRI and BLI data were combined to show that cells were transplanted at the correct site and that they remained viable and present in a detectable amount. The MRI enabled visualization of ferumoxytol at the transplantation site after 48 days and we were able to observe changes in the hypointense regions indicating migration of cells, most likely HFBSCs as indicated by Perls’ Prussian blue staining with DAB intensification for iron oxide in cryosections, towards the area of TBI (Fig. 3b′, day 48, arrow). This is in line with the finding of Zheng et al. who were able to image neural stem cells for up to 87 days in vivo (Zheng et al. 2015).

It is of importance to mention that it is not possible to correlate both modalities, BLI and MRI. Therefore, we used BLI for quantitative data and MRI was applied to gain anatomical information of the site of injection. In this way, we took advantage of the anatomical resolution achievable with MRI as well as the sensitivity of the BLI signal, together with information about gene expression. With respect to the MRI data, we only acquired T2*-weighted scans with a single echo. In a recent study, Mishra et al. used multi-echo MRI scans to measure the iron oxide load of MSCs (Mishra et al. 2018). However, it was not possible to reproduce this analysis due to the scan settings used in our study. Moreover, the resolution of the MRI is much lower than the size of the cells. This means that also migration and dispersion of the cells from the local injection volume to a much wider volume will result in a different sub-voxel distribution of iron, again resulting in changes in T2* relaxation that do not reflect true iron quantification. Therefore, we do not consider the quantification of the T2* relaxation as a precise measure of the iron oxide load of cells.

Transduced HFBSCs maintained fluorescence after fixation, sectioning and (immune)staining. Therefore, we were able to visualize the copGFP expressed by HFBSCs in cryosections of mouse brains. We verified that this green fluorescence was obtained from copGFP and excluded auto-fluorescence by staining the sections with primary antibodies against either copGFP or Luc2, which were both expressed by the transplanted cells. The transplanted HFBSCs showed staining for the neural marker neurofilament (NF-Pan), which is in line with previous findings (Ladewig et al. 2008; Tennstaedt et al. 2015) but not for glial fibrillary acidic protein (GFAP), which indicates their differentiation towards a neuronal lineage. In addition, the majority of copGFP-expressing HFBSCs in brain sections of TBI mice were positive for nestin, a marker for NCSCs and neural progenitors, underlining the neural differentiation potential of these stem cells. However, we can only speculate about their phenotype when fully matured. We assume that HFBSCs, similar to the skin-derived precursor cells, which are also derived from the neural crest, would differentiate into peripheral neurons (Fernandes et al. 2006; Fernandes et al. 2004). Staining for DCX, a marker that is expressed early on in neural differentiation, was negative, which confirmed our bioluminescence results that, at this time point the cells passed their commitment of neuronal differentiation and progressed towards a more mature neuronal state. Underlining this finding was the fact that the transplanted cells were not positive for cell proliferation marker Ki-67, indicating that HFBSCs did not enter a harmful, tumorigenic state of uncontrolled proliferation. Surprisingly, transplanted cells showed positive staining for the extracellular matrix proteins laminin (all) and fibronectin (some). Laminin and fibronectin have been shown to play important roles in neurite guidance regeneration of tissue after TBI and motility of neural crest cells, which further support the potential role of HFBSCs in tissue regeneration in TBI (Evans et al. 2007; Strachan and Condic 2008; Tate et al. 2007).

After identifying transplanted HFBSCs within brain sections of TBI mice, we stained these sections for Fe^3+^, a component of ferumoxytol. The staining revealed that HFBSCs still contained ferumoxytol after 58 days in vivo. Our results indicate that ferumoxytol was partially excreted by HFBSCs and taken up by cells in the surrounding tissue, e.g., macrophages. However, a substantial part of the hypointense signal on the MRI originates from ferumoxytol-containing HFBSCs.

Moreover, our finding of fluorescent cells, which are localized in the corpus callosum and the site of TBI (in the superficial cortex) in brain sections, may indicate that HFBSCs participate in recovery after neural trauma. The mechanism of how this recovery might occur remains unknown. However, the need to further elucidate potential recovery persists. Recovery may occur by cell replacement due to differentiation, through paracrine effects, or combination of both. In case both mechanisms occur, a strategy could be to transplant cells in biologic scaffolds made of brain-derived extracellular matrices in the form of hydrogels with the aim to enhance survival and promote angiogenesis (Crapo et al. 2014). On the other hand, if the paracrine effect prevails, a therapeutic strategy based on the sole administration of exosomes derived from HFBSCs, it would represent an alternative neurorestorative therapy. Moreover, the exosomes could be tailored to maximize clinical benefits (Zhang et al. 2019).

## Conclusion

In conclusion, this first study about HFBSCs transplantation in a mouse model of TBI indicates that HFBSCs are able to survive in mice brain and the procedure is safe in mice. In this relative short time, most of the transplanted cells remained nestin positive but some individual cells undergo differentiation towards a neuronal cell lineage and a part could be found in the lesioned area. Because of all this evidence, HFBSCs qualify as possible candidates for cell-based therapy of TBI, although further studies are required to elucidate their ability to enhance repair processes and improve cognitive function.

## Electronic supplementary material

ESM 1 (DOCX 12 kb)

ESM 2 (PNG 323 kb)
