# Supplementary material for: Multimodal imaging of hair follicle bulge-derived stem cells in a mouse model of traumatic brain injury
Source: Cell Tissue Res. 2020 Feb 8;381(1):55–69. doi: 10.1007/s00441-020-03173-1 (PMC7306043; doi:10.1007/s00441-020-03173-1)
Supplement: Supplementary file 1 — (DOCX 12 kb) [file 441_2020_3173_MOESM1_ESM.docx]

**Figure S1. Control stainings.** Positive immunostaining for laminin inside of HFBSCs as well as the control cell lines HDFa and RT4-D6PT2 (gray). These stainings provide a similar pattern to the staining observed in the brain tissue sections. A specific staining for Luc2 can be observed in transduced HFBSCs, but HDFa and RT4-D6PT2 do not stain for Luc2 (red). The native fluorescence of copGFP is visible in transduced HFBSCs (green), which is absent in the non-transduced control cell lines.
