# Supplementary figures and images for: Multimodal imaging of hair follicle bulge-derived stem cells in a mouse model of traumatic brain injury
Source: Cell Tissue Res. 2020 Feb 8;381(1):55–69. doi: 10.1007/s00441-020-03173-1 (PMC7306043; doi:10.1007/s00441-020-03173-1)

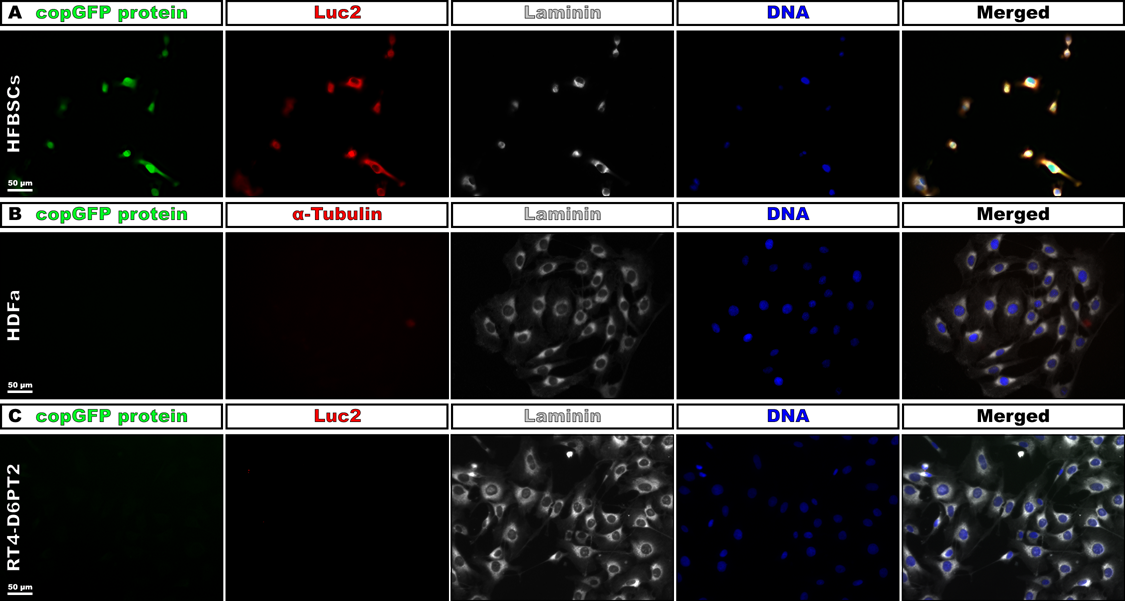

Supplement: Supplementary file 2 — (PNG 323 kb) [file 441_2020_3173_MOESM2_ESM.png]
